# Proteolytic Rafts for Improving Intraparenchymal Migration of Minimally Invasively Administered Hydrogel-Embedded Stem Cells

**DOI:** 10.3390/ijms20123083

**Published:** 2019-06-24

**Authors:** Marcin Piejko, Anna Jablonska, Piotr Walczak, Miroslaw Janowski

**Affiliations:** 1 Russell H. Morgan Department of Radiology and Radiological Science, Division of MR Research, The Johns Hopkins University School of Medicine, Baltimore, MD 21231, USA; marcin.piejko@gmail.com (M.P.); anna.m.jablonska@gmail.com (A.J.); pwalczak@mri.jhu.edu (P.W.); 2 Cellular Imaging Section and Vascular Biology Program, Institute for Cell Engineering, The Johns Hopkins University School of Medicine, Baltimore, MD 21231, USA; 3 3rd Department of General Surgery, Jagiellonian University Medical College, 31202 Krakow, Poland

**Keywords:** hydrogel, rafts, enzymes, immobilization, cell transplantation

## Abstract

The physiological spaces (lateral ventricles, intrathecal space) or pathological cavities (stroke lesion, syringomyelia) may serve as an attractive gateway for minimally invasive deployment of stem cells. Embedding stem cells in injectable scaffolds is essential when transplanting into the body cavities as they secure favorable microenvironment and keep cells localized, thereby preventing sedimentation. However, the limited migration of transplanted cells from scaffold to the host tissue is still a major obstacle, which prevents this approach from wider implementation for the rapidly growing field of regenerative medicine. Hyaluronan, a naturally occurring polymer, is frequently used as a basis of injectable scaffolds. We hypothesized that supplementation of hyaluronan with activated proteolytic enzymes could be a viable approach for dissolving the connective tissue barrier on the interface between the scaffold and the host, such as pia mater or scar tissue, thus demarcating lesion cavity. In a proof-of-concept study, we have found that collagenase and trypsin immobilized in hyaluronan-based hydrogel retain 60% and 28% of their proteolytic activity compared to their non-immobilized forms, respectively. We have also shown that immobilized enzymes do not have a negative effect on the viability of stem cells (glial progenitors and mesenchymal stem cells) in vitro. In conclusion, proteolytic rafts composed of hyaluronan-based hydrogels and immobilized enzymes may be an attractive strategy to facilitate migration of stem cells from injectable scaffolds into the parenchyma of surrounding tissue.

## 1. Introduction

In recent years, we have been witnessing unprecedented progress in stem cell biology as well as in deciphering pathophysiology of diseases. As a consequence, access to highly potent stem cell populations is improving, and attractive therapeutic targets are being defined. However, the efficacy of widespread stem cell delivery has lagged behind basic science and clinical opportunities, delaying momentum for a massive “bench to bedside” translation in the field of regenerative medicine. Body cavities are an attractive gateway for stem cell delivery as they typically allow for wide biodistribution in the receiving organs. Indeed, stem cell delivery to various body cavities, such as the peritoneum, pleura [1], pericardium [2], or post-stroke cavity [3], have been attempted, although no significant transmigration of transplanted cells to the host tissues was observed. Particularly, neurodegenerative disorders carry a grim prognosis, with the blood–brain barrier being a factor that limits access to the central nervous system. Intra-arterial route is an attractive, minimally invasive method for cell delivery to the brain [4,5]; however, targeting the spinal cord is still challenging due to its complex vascular anatomy. Until now, direct needle-based injection to parenchyma has been the most frequently used method of stem cell delivery to the spinal cord, but high level of invasiveness and restricted biodistribution are limiting factors [6]. In contrast, injection to the intrathecal space prevents potential direct injury to the organ due to the volume of transplanted cells and vehicle [7] and prevents cell damage due to the pressure of parenchyma and immune response, which can be potentiated by the hostile environment of disease-damaged tissue [8]. Due to the high surface-to-volume ratio and easy access, intrathecal transplantation might be a viable strategy for cell delivery in diseases affecting the spinal cord, such as amyotrophic lateral sclerosis [9]. Unfortunately, till this day, there have been no reports of effective transmigration of intrathecally delivered cells into the spinal cord in adult organisms. 

Glial progenitors (GPs) transplanted into the lateral ventricle have been shown to be capable of rescuing the lifespan of dysmyelinating mice. The therapeutic benefit, however, was found to be highly dependent on their widespread distribution throughout the host brain [10]. Thus, wide dissemination of GPs is essential for successful translation of glia replacement therapy [11]. Broader biodistribution of intrapericardially [12] and intrapleurally [1] injected mesenchymal stem cells (MSCs) is also desired. Taken together, to fully exploit the potential of body cavities as a route for cell transplantation, there is an urgent need to improve the migration of stem cells to the parenchyma of target organs. Injectable scaffolds have been widely tested as a structural and trophic support for stem cells injected into cavities [1]. Scaffolds also introduce new, unexplored opportunities for enhancing transmigration of stem cells from cavities to target organs. Here, we present an initial assessment of feasibility of a new approach based on incorporating proteolytic rafts into hydrogel scaffold to facilitate migration of glial progenitors and mesenchymal stem cells from body cavities to the parenchyma of target organs. 

## 2. Results

### 2.1. Enzymatic Activity of Immobilized Trypsin and Collagenase in Hyaluronan-Based Hydrogels

**Figure 1:**
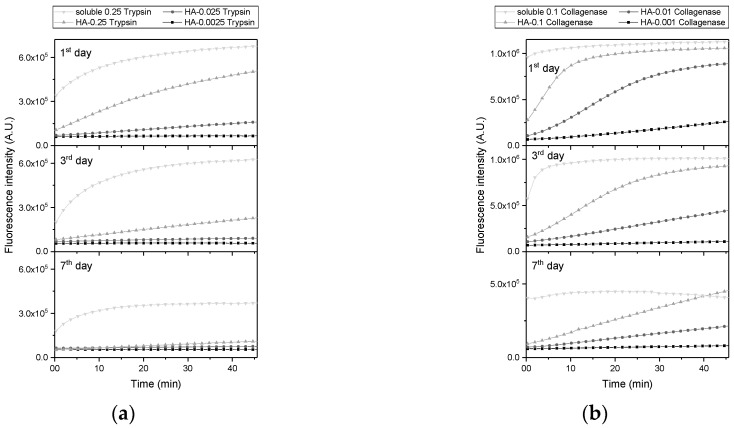
Kinetic curves of trypsin (**a**) and collagenase (**b**) immobilized in hydrogel revealed leading-type kinetics for both enzymes.

Trypsin and collagenase derived by sulfo-SMCC (sulfosuccinimidyl 4-[N-maleimidomethyl]cyclohexane-1-carboxylate) (Thermo, USA) and immobilized into hydrogels maintained proteolytic activity, as demonstrated by the fluorescein-labeled collagen assay (a substrate of EnzCheck assay) (Figure 1). Collagenase immobilized in hydrogels displayed higher enzymatic activity, as expressed by the higher fluorescence intensity at 30 min of 1.0 × 10^6^ A.U. for hyaluronan acid (HA)-0.1 mg/ml collagenase in comparison to 6.0 × 10^5^ A.U. for HA-0.25 mg/mL trypsin. The lower specificity of native trypsin against collagen, in combination with the same diffusional barrier of collagenase- and trypsin-immobilized hydrogels, explains the lower enzymatic activity (fluorescence intensity) in tryptic hydrogels. Kinetic curves of trypsin immobilized in hydrogels (Figure 1a) and collagenase activity after one day of incubation (Figure 1b) characterized typical leading-type reaction, which allowed us to apply EnzCheck assay to our proof-of-concept study. 

**Figure 2:**
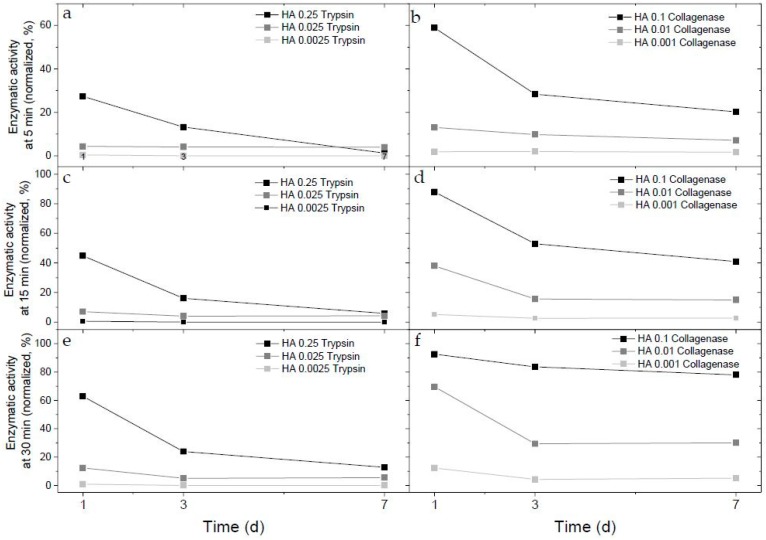
Evolution of trypsin (**a**,**c**,**e**) and collagenase (**b**,**d**,**f**) activity immobilized into hyaluronan-based hydrogels with no cells. Enzymatic activity was defined as a change in fluorescence intensity during 5 min (**a,b**), 15 minutes (**c,d**), and 30 min (**e,f**) of EnzCheck assay. Enzyme–hydrogel constructs were incubated for 7 days at 37 °C, 5% CO_2_, and 90% humidity. Normalization was performed to equivalent of native enzymes.

Kinetic curves were normalized to equivalent portion of native trypsin or collagenase. We selected three time points for further assessment of enzymatic activity with cells: 5 min to estimate normalized enzymatic activity; 15 min, which is the middle of the slope; and 30 min at the plateau phase (Figure 2). 

The enzymatic activity normalized for the 5-min interval decreased over time for all enzymatic hydrogels. This means that immobilized trypsin and collagenase lost their activity with time. On the seventh day of experiment, the activity remained at only 33.3% and 6.7% of the initial rate of HA-0.1 mg/mL collagenase and HA-0.25 mg/mL trypsin, respectively. On day 7, the 30-min reaction with HA-0.1 mg/mL collagenase digested around 84% of the EnzCheck substrate relative to the day 1 activity. In the same testing regime, HA-0.25 mg/mL trypsin maintained only 20% of the initial activity. The initial enzymatic activity of 0.01 and 0.001 HA-collagenase was 13% and 2%, respectively, and remained low on the third day at 10% and 2%, respectively. On day 7, activity went down to 7% for HA-0.01 mg/mL collagenase and 1.7% for HA-0.001 mg/mL collagenase. Lower percentage of HA-trypsin hydrogels maintained 4% of activity on days 1, 3, and 7 for HA-0.025 mg/mL trypsin and was undetectable for HA-0.0025 mg/mL trypsin. This comparison of enzymatic activity clearly reveals that immobilized collagenase is more stable than immobilized trypsin. Based on kinetic experiments, we selected 0.25 mg/mL of trypsin and 0.1 mg/mL of collagenase (final concentration in hydrogel) for cell embedding studies with measurements of enzymatic activity and cell viability. 

### 2.2. Enzymatic Activity Measurements for Hydrogel-Embedded Stem Cells 

**Figure 3:**
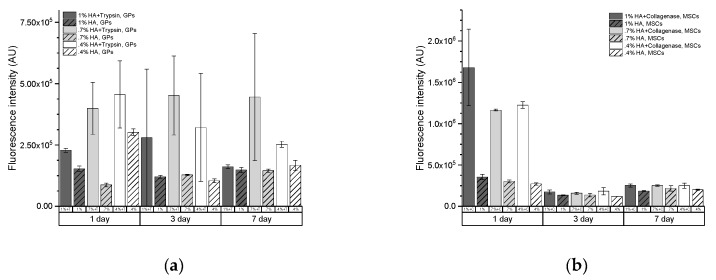
Activity of immobilized 0.25 mg/mL trypsin (**a**) and 0.1 mg/mL collagenase (**b**) (non-patterned bars) in comparison to their no-enzyme controls (patterned bars) were assessed in hydrogels with embedded cells.

Multivariate regression revealed that the tryptic activity was a major source of variability (F = 35.12, *p* < 0.001) in HA embedded with GPs, while HA concentration had a much lower but still significant impact (F = 3.82, *p* = 0.032). An interaction between tryptic activity and HA concentration was also observed (F = 4.87, *p* = 0.0138), while time itself was not a source of significant variability (Table A2) (Figure 3). 

In contrast, time was the most substantial source of variability for HA embedded with MSCs (F = 203, *p* < 0.001), which was only slightly higher than the collagenase activity (F = 157, *p* < 0.001, Table A1) and the interaction of time and collagenase activity (F = 121.78, *p* < 0.001) (Table A1). Hydrogel as well as the interaction of time and hydrogel was a lower but still statistically significant source of variability (F = 3.96, *p* = 0.0284, and F = 4.26, *p* = 0.0067, respectively). Therefore, enzymatic activity was observed in both experiments. However, collagenase activity faded over time, which was not the case for tryptic activity. Interestingly, HA concentration positively correlated with the activity of collagenase, while it negatively correlated with tryptic activity (Table A2) (source data are presented in Table A5).

### 2.3. In Vitro Cell Viability in Enzymatic Hydrogels

**Figure 4:**
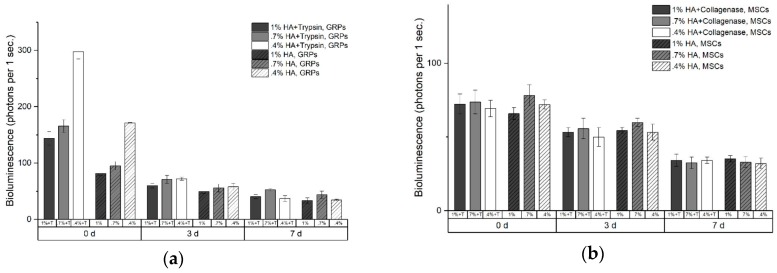
Cells viability in tryptic (**a**) and collagenase (**b**) rafts measured as bioluminescence signal of glial progenitors (GPs) and mesenchymal stem cells (MSCs) for 7 days.

Multivariate analysis revealed positive effect of trypsin on GP viability (F = 466.8, *p* < 0.001), while cell survival faded over time (F = 2005.2, *p* < 0.001) and negatively correlated with HA concentration (F = 251.43, *p* < 0.001) (Figure 4, Table A4). Survival of MSCs also faded over time (F = 306.49, *p* < 0.001, Table A3), while both collagenase activity and HA concentration did not affect MSC viability (F = 0.58, *p* = 0.45 and F = 3.20, *p* = 0.053, respectively). No interactions between variables were observed. 

## 3. Discussion

Immobilization of enzymes is a well-known area of industrial biotechnology [13,14,15,16]. At present, there are several techniques that use immobilization of relatively expensive enzymes in solid phase of carriers to operate in areas of biotransformation, food processing, medical diagnostics, and pharmacy [15]. In the field of cellular therapies, Deller et al. modified cellular membrane of MSCs by thrombin immobilization to improve cell adhesion, homing, and resilience to hypoxia [17]. Functionalization of biomaterials opens a new possibility to improve cell transplantation. The key aim of this study was to provide evidence that enzymes immobilized on activated hyaluronan maintain their proteolytic activity and are not detrimental to embedded stem cells.

Here, we presented a proof-of-concept study showing the potential utility of proteolytic rafts composed of immobilized enzymes and hydrogel scaffolds that could be used for enhancing cell migration from body cavities to the target organs. Such feature is evidently needed based on previous cell transplantation studies [4,5,6]. We demonstrated that the introduction of trypsin or collagenase, two proteolytic enzymes, to hyaluronic acid-based hydrogel could facilitate partial digestion of the connective tissue typically separating cavities from organs. Importantly, we showed that enzymatic rafts had no negative effect on the survival of embedded cells, and tryptic activity even facilitated survival of GPs. In contrast, collagenase activity was neutral for MSCs. We also observed a negative correlation between HA concentration and GP viability, which may indicate that GPs better tolerate lower stiffness, while HA concentration and immobilization of enzymes did not affect survival of MSCs. This phenomenon might be related to the in vivo natural cell microenvironment with much less stiffness of the brain tissue, which is home to GPs, in comparison to MSCs inhabiting connective tissue. It is also possible that higher stiffness might negatively affect MSCs, but this would be beyond the range of viscoelasticity observed in our experiment.

Various procedures related to stem cell handling, such as isolation or transplantation, are strongly associated with stressful factors like ionic, oncotic, and mechanical disturbances and changes in extracellular matrix (ECM) composition [18]. This is why delivery of cells embedded in hydrogels that can partially replace missing ECM is a very promising strategy [19,20]. In our study, we used hyaluronan-based hydrogels. However, similar to Mohand-Kaci et al. [21], we observed poor cell viability, which was additionally correlated with the HA concentration in case of GPs. Nevertheless, the 0.4%-HA hydrogels are mechanically labile and difficult to use in a clinical setting. For this reason, it is necessary to introduce new solutions that can increase survival and migration of cells transplanted in hydrogels with higher content of hyaluronic acid. 

In our study, the tryptic and collagenase proteolytic capability to digest ECMs was detected in all time points; however, a decrease of this activity over time was visible. Self-limiting enzymatic activity over time plays a pivotal role in the prevention of uncontrolled off-target proteolysis. However, persistent tryptic activity was observed on days 3 and 7 in the presence of GPs, unlike the low activity in HA with no cells. Hydrogel with no cells consist of trypsin attached to HA via relatively long spacer as SMCC. Consequently, trypsin has no substrate to digest, and autolysis may dominate. In the presence of cells, trypsin has access to a rich mixture of peptides and proteins released by GP or from culture medium, so autolysis of trypsin may be less expressed. 

A decrease in collagenase activity observed on day 3 in MSC-embedded, but not in control, hydrogels may be explained by the presence of tissue inhibitors of metalloproteinases (TIMPs) in MSCs, which can mitigate activity of collagenase [22]. The only cells expressing tryptic activity inhibitors (i.e., alpha-1-antitrypsin) are hepatocytes [23]. As expected, a similar phenomenon was not observed in hydrogels with immobilized trypsin and embedded GPs. This data shows a need for careful optimization of enzymatic raft formulation as unpredictable balance between enzymes and their endogenous inhibitors can lead to side effects. 

With the bioluminescence viability study, we showed that enzymatic activity in hydrogels has no negative effect on the survival of GPs and MSCs. Even more, our in vitro and in vivo data showed slightly beneficial effect of trypsin-HA on survival of GPs. This observation is contrary to the common experience about the detrimental effect of prolonged exposition of cells to proteolytic enzymes, mainly trypsin, during subculturing [24,25,26]. This discrepancy can be explained by the fact that enzymes immobilized on HA scaffold have limited possibility to be up-taken by the cells. Also, as shown earlier, immobilized enzymes could have decreased activity in comparison to their activity in solution [27,28]. The beneficial effect of proteolytic rafts on cells survival, however small, is very promising and shows a need for further studies in this field, including new composition of enzymes and their concentrations. 

Limitations: We performed proof-of-concept in vitro study, but in vivo validation of our concept needs to be performed in future. We have also not performed rheometry, so we do not know the exact impact of HA concentration and immobilization of enzymes on the viscoelastic properties of hydrogels. This knowledge might be necessary to be acquired prior to further hydrogel optimization to increase cell survival and proliferation.

## 4. Materials and Methods 

### 4.1. Materials

Cell culture medium and supplements were obtained from Gibco, Thermo Fisher Scientific, (Waltham, MA, USA). HyStem components (Glycosil and Extralink) were purchased in EsiBio (Alameda, California, USA). EnzCheck collagenase kit was obtained from Molecular Probes (Eugene, OR, USA). All chemicals were purchased from Sigma-Aldrich (St. Louis, Missouri, USA). 

### 4.2. Cell Harvest and Culture 

GPs and MSCs were harvested from transgenic mice expressing green fluorescence protein (GFP) under myelin proteolipid protein (PLP) promoter (GFP expression induced by mature oligodendrocytes) and luciferase (luc) under beta-actin promoter (stable expression). Harvesting of GPs was conducted as previously described [10], and subsequent cell culture was performed in a medium consisting of DMEM/F12 supplemented with N2 and B27, bovine serum albumin (BSA), heparin, and bFGF. GPs were cultured on polystyrene flask coated with poly-L-lysine and laminin. MSCs were harvested as described [29] and maintained in low-glucose DMEM/F12 supplemented with 10% fetal bovine serum (FBS) and 1% penicillin/streptomycin. MSCs were plated on polystyrene flasks with no coating agents. Media were exchanged every third day. At 70%–80% confluence, 0.05% trypsin/ethylenediaminetetraacetic acid (EDTA) was used to passage the cells. Cells between the 2nd and 4th passage were used for the experiments. 

### 4.3. Trypsin and Collagenase Derivation

Bifunctional amin-to-sulfhydril crosslinker (sulfosuccinimidyl 4-[N-maleimidomethyl]cyclohexane-1-carboxylate (sulfo-SMCC) (Thermo, USA) was chosen to make enzymes a crosslinking partner for activated hyaluronan. In brief, approximately 2.5 mg/mL of trypsin or 10 mg/mL of collagenase were prepared in solution consisting of 5× PBS and 1 mM EDTA, pH 7. Immediately, 100 µL of 10 mM solution sulfo-SMCC in DMSO was added to 1 mL of trypsin or 1 mL of collagenase. Modification of N-end of enzymes was carried out for up to 30 min in 25 °C with 1000 rpm agitation. Reaction was stopped by desalting on ZebaTM Spin columns, 7K MWCO. Finally, 2.5 mg/mL trypsin-MCC and 10 mg/mL of collagenase-MCC in 1× PBS were stored at −20 °C. 

### 4.4. Hydrogel Preparation

Hydrogels were prepared from HyStem (EsiBio, USA) components: 10 mg/mL thiol-modified hyaluronan acid and 10 mg/mL of poly-(ethylene glycol) diacrylate (PEGDA) dissolved in PBS. Hydrogels were prepared according to the manufacturer’s manual. To form a nonenzymatic raft, 100 µL of HA were mixed with 25 µL of PEGDA. To prepare an enzymatic raft, 90 µL of 1% HA was mixed with 10 µL of trypsin-MCC or 10 µL of collagenase-MCC, and 25 µL of PEGDA was then added. For viability assessment immediately before PEGDA addition, 10 µL of MSC or GP suspension was transferred to the solution of HA or HA/enzymes-MCC. Whole mixtures were pipetted 3X, and 30 µL was transferred to 3 wells of 96-well plates. 

### 4.5. Enzymatic Activity Assay

To assess enzymatic activity, EnzCheck collagenase activity kit was used. Assays were performed according to the manufacturer protocol. All measurements were conducted with 30 µL of hydrogel to 30 µL of fluorescence substrate. To optimize reaction and to check the compatibility of the EnzCheck assay with tryptic activity, kinetic of digestion was performed. Hydrogels with collagenase or trypsin were prepared in 96-well black plates. Two hours, 2 days, and 7 days after cross-linker addition, hydrogels were washed 3× with PBS. To normalize data, a fresh control solution consisting of 10 µL of 10 mg/mL collagenase-MCC or 2.5 mg/mL trypsin-MCC with 90 µL of PBS was prepared before each measurement. Then, 25 µL of PEGDA was added into collagen or trypsin mix, and solutions were divided into 3 parts of 30 µL each. Then, 30 µL of fluorescein-labeled collagen was transferred to each well with 30 µL of solid hydrogel, and fluorescence emission was immediately acquired using Victor 3 plate reader (PerkinElmer, Boston, MA, USA) (excitation filter 485 nm, emission filter 535 nm, 37 °C). After 45 min, substrate was dropped out, and hydrogels were washed 3× with PBS and kept in incubator until the next measurements. All data were normalized to digestion kinetic of trypsin-MCC or collagenase-MCC. 

Enzymatic activity assay of hydrogels with embedded cells were carried out on the same plate as viability assay but at 6, 24, 48 h, and 7-day time points. Before substrate addition, hydrogels were washed 3× with PBS. Then, substrate was incubated with hydrogels up to 25 min at 37 °C, and 30 µL of the above post-reaction mixture and the hydrogels were transferred to the 96-well black plate, diluted 2× by PBS, and fluorescence was acquired. Plates with hydrogels were washed 3× with warm cell culture medium and stored at 37 °C for subsequent measurements.

### 4.6. In Vitro Viability Assessments

Viability of luciferase-positive MSCs and GPs embedded in hydrogels was assessed by bioluminescence measurements. To address the problem of low viability of cells embedded into manufacturer-recommended 1% formulation HA hydrogels [21,30], HA concentration was reduced to 0.7% and 0.4%. GP suspension (10 µL / 10 mln/mL) in cell culture medium was transferred to 100 µL of 1%, 0.7%, or 0.4% HA (control) or a mixture consisting of 90 µL of various percentage of HA (1%, 0.7%, or 0.4% HA in PBS) and 10 µL of 2.5 mg/mL trypsin-MCC (tryptic raft). Similarly, 10 µL of 1 mln/mL MSC suspension was mixed with control HA and a mixture consisting of 90 µL of HA and 10 µL of 10 mg/mL collagenase-MCC (collagenase raft). Finally, 100 µL of activated hyaluronan mixture was cross-linked with 25 µL of PEGDA and immediately placed in triplicate (per 30 µL) on the white 96-well plate with a clear bottom (Greiner, Monroe, North Carolina, USA). Ten minutes later, 30 µL of warm, balanced cell culture medium was gently transferred on the top of the forming hydrogels, which were incubated overnight at 37 °C, 5% CO_2_. Twenty hours later, the first measurement of viability was done. Before addition of D-luciferin, hydrogels were washed 3× by warm medium. Then, 70 µL of 150 µg/mL D-luciferin in cell culture medium was transferred, and bioluminescence was acquired (3 times, acquisition time: 3 s per well, 37 °C). After measurements, the medium with D-luciferin was removed, and hydrogels were washed 3× with cell culture medium. Enzymatic activity assays were performed on the same cells 6 h later. Plates were maintained in cell culture incubator, and subsequent measurements of bioluminescence were performed on days 2 and 7. 

### 4.7. Statistics

All statistical calculations were performed using SAS 9.4. Multivariate regression were expressed as type III test of fixed effects, and comparison between the means was performed using least square means (LSM) embedded within PROC MIXED. 

## 5. Conclusions

Proteolytic rafts may be an attractive approach to improve penetration of transplanted stem cells from body cavities to the parenchyma of target organs. Importantly, no negative effects of proteolytic enzymes on cell viability were observed. 

## Abbreviations

HA	activated hyaluronan acid
PEGDA	poly(ethylene glycol) diacrylate
GRPs	glial-restricted progenitors
MSC	mesenchymal stem cells
Sulfo-SMCC	sulfosuccinimidyl 4-[N-maleimidomethyl]cyclohexane-1-carboxylate

## Appendix A

**Table A1:** Statistical analysis: Collagenase activity in HA embedded with MSCs.

Type 3 Tests of Fixed Effects				
	Num DF	DenDF	*F* Value	Pr > F
Time	2	34	203.48	<0.0001
Enzyme	1	34	157.37	<0.0001
Hydrogel	2	34	3.96	0.0284
Time*Enzyme	2	34	121.78	<.0001
Time*Hydrogel	4	34	4.26	0.0067
Enzyme*Hydrogel	2	34	2.81	0.0745
Time*Enzyme*Hydrogel	4	34	2.28	0.0804

**Table A2:** Statistical analysis: Collagenase activity in HA embedded with GPs.

Type 3 Tests of Fixed Effects				
	Num DF	DenDF	*F* Value	Pr > F
Time	2	34	0.98	0.3868
Enzyme	1	34	35.12	<0.0001
Hydrogel	2	34	3.82	0.0320
Time*Enzyme	2	34	0.90	0.4173
Time*Hydrogel	4	34	2.04	0.1113
Enzyme*Hydrogel	2	34	4.87	0.0138
Time*Enzyme*Hydrogel	4	34	0.13	0.9687

**Table A3:** Statistical analysis: Viability of MSCs.

Type 3 Tests of Fixed Effects				
	Num DF	DenDF	*F* Value	Pr > F
Time	2	34	306.49	<0.0001
Enzyme	1	34	0.58	0.4534
Hydrogel	2	34	3.20	0.0533
Time*Enzyme	2	34	0.55	0.5843
Time*Hydrogel	4	34	1.74	0.1634
Enzyme*Hydrogel	2	34	1.07	0.3545
Time*Enzyme*Hydrogel	4	34	0.85	0.5011

**Table A4:** Statistical analysis: Viability of GPs.

Type 3 Tests of Fixed Effects				
	Num DF	DenDF	*F* Value	Pr > F
Time	2	34	2005.19	<0.0001
Enzyme	1	34	466.80	<0.0001
Hydrogel	2	34	251.43	<0.0001
Time*Enzyme	2	34	246.87	<0.0001
Time*Hydrogel	4	34	239.29	<0.0001
Enzyme*Hydrogel	2	34	15.31	<0.0001
Time*Enzyme*Hydrogel	4	34	18.16	<0.0001

**Table A5:** Enzymatic activity of the rafts.

	HA	Fluorescence (A.U.)		
		1st day	3rd day	7th day
**HA-1 mg/mL collagenase**	1.0% + C	1.68 × 10^6^	1.74 × 10^5^	2.52 × 10^5^
	1.0%	3.55 × 10^5^	1.35 × 10^5^	1.86 × 10^5^
	0.7% + C	1.16 × 10^6^	1.58 × 10^5^	2.52 × 10^5^
	0.7%	3.02 × 10^5^	1.37 × 10^5^	2.16 × 10^5^
	0.4% + C	1.22 × 10^6^	1.82 × 10^5^	2.49 × 10^5^
	0.4%	2.71 × 10^5^	1.21 × 10^5^	2.01 × 10^5^
**HA-0.25 mg/mL trypsin**	1.0% + T	2.29 × 10^5^	2.80 × 10^5^	1.61 × 10^5^
	1.0%	1.53 × 10^5^	1.20 × 10^5^	1.48 × 10^5^
	0.7% +T	3.99 × 10^5^	4.52 × 10^5^	4.46 × 10^5^
	0.7%	8.73 × 10^4^	1.28 × 10^5^	1.46 × 10^5^
	0.4% + T	4.56 × 10^5^	3.21 × 10^5^	2.53 × 10^5^
	0.4%	3.02 × 10^5^	1.04 × 10^5^	1.67 × 10^5^
